# OntoloViz: a GUI for interactive visualization of ranked disease or drug lists using the MeSH and ATC ontologies

**DOI:** 10.1093/bioadv/vbad113

**Published:** 2023-08-23

**Authors:** Matthias Ley, Andreas Heinzel, Lucas Fillinger, Klaus Kratochwill, Paul Perco

**Affiliations:** Computational Biology Department, Delta4 GmbH, Vienna 1080, Austria; Division of Pediatric Nephrology and Gastroenterology, Department of Pediatrics and Adolescent Medicine, Comprehensive Center for Pediatrics, Medical University Vienna, Vienna 1090, Austria; Computational Biology Department, Delta4 GmbH, Vienna 1080, Austria; Department of Internal Medicine III, Medical University Vienna, Vienna 1090, Austria; Computational Biology Department, Delta4 GmbH, Vienna 1080, Austria; Computational Biology Department, Delta4 GmbH, Vienna 1080, Austria; Division of Pediatric Nephrology and Gastroenterology, Department of Pediatrics and Adolescent Medicine, Comprehensive Center for Pediatrics, Medical University Vienna, Vienna 1090, Austria; Computational Biology Department, Delta4 GmbH, Vienna 1080, Austria; Department of Internal Medicine IV, Medical University Innsbruck, Innsbruck 6020, Austria

## Abstract

**Motivation:**

Structured vocabularies for drugs and diseases represent, besides their primary use for annotating scientific literature or scientific information in general, a valuable resource for visualizing aggregated information. The Medical Subject Headings (MeSH) and Anatomical Therapeutic Chemical (ATC) ontologies are widely used structured vocabularies for diseases and drugs, respectively. Their hierarchical tree-like structure can be used as a basis for creating intuitive visual displays for specific diseases and drugs within their higher-order classifications. Such displays are helpful means to contextualize diseases and drugs in various settings such as in drug repositioning. However, there are few tools that can harness the potential of these structured ontologies to create informative visual representations without extensive programming and data processing skills.

**Results:**

We have developed OntoloViz, a Graphical User Interface (GUI) for visualizing annotated lists of drugs or diseases in the context of their MeSH or ATC ontologies in an intuitively interpretable sunburst layout. Minimum input is a list of disease or drug names. Users in addition have the option to specify numerical parameters for the input lists to enhance the visualization, e.g. to visualize term frequencies. The GUI allows values to be propagated upwards in the respective ontology tree structure thus facilitating exploration of gene and drug lists. We present two use cases for OntoloViz, namely (i) a graphical representation of clinically tested drugs for coronavirus disease (COVID-19) based on ATC Classification and (ii) a graphical representation of literature annotation of human diseases on the MeSH ontology.

**Availability and implementation:**

The OntoloViz package can be retrieved from PyPi. The source code along with test data, template, and documentations are available at GitHub (https://github.com/Delta4AI/OntoloViz).

## 1 Introduction

Disease and drug ontologies are key elements in biomedical and clinical research. They are used for example to index biomedical literature thus facilitating literature mining approaches. Commonly used disease ontologies include the World Health Organization’s International Classification of Diseases (ICD) (World Health Organization 1993), the Human Disease Ontology (Schriml *et al.* 2022), developed at the EMBL-EBI, or the MeSH controlled vocabulary thesaurus (Lipscomb 2000). The Anatomical Therapeutic Chemical (ATC) classification is a widely used structured vocabulary for drugs. Disease ontologies are valuable tools that aid contextualization and interpretation of results in exploratory analyses as for example in large compound screenings or computational drug repositioning.

Drug repositioning aims to identify novel uses for already approved drugs. Drug repositioning gained a major boost during the COVID-19 pandemic, which has led to a tsunami of drug repositioning approaches to expedite the discovery and development of effective treatment options for COVID-19 and associated complications (Mucke 2020). A large number of drugs belonging to a broad array of classifications have been experimentally tested during that time, ranging from antiviral drugs to anti-inflammatory agents. We will use this example as a use case to illustrate how OntoloViz can be employed to create a structured overview of diverse drug landscapes. In the context of indication expansion on the other hand, i.e. finding additional indications of interest for an approved drug or a drug in development, the visualization of disease candidates on the level of disease ontologies supports finding commonalities and detecting patterns and trends regarding the potential usefulness of the drug in certain disease areas.

Sunburst plots are widely used to graphically display hierarchical data in a structured way. We decided to use sunburst plots within OntoloViz as the hierarchical structure is a lot clearer than in treemap visualizations but on the other hand they are also more compact than for example icicle plots. Even though there are R packages and Python libraries available to generate generic sunburst plots, these packages require programming and data handling experience to create desired and visually appealing outputs. A dedicated easy-to-use application for the visualization of diseases or drugs in the context of their classification has been lacking so far. To fill this gap, we have developed OntoloViz, a Graphical User Interface (GUI) for interactive visualization of ranked disease or drug lists based on widely used ontologies, allowing biomedical researchers without programming experience to explore data from large disease or drug screenings on the level of structured ontologies.

## 2 Materials and methods

### 2.1 Installation and dependencies

OntoloViz can be run by downloading the latest release for Windows available at https://github.com/Delta4AI/OntoloViz/releases/latest. This GitHub repository also contains downloadable templates, test data, and example plots. In addition to the standard Python library, OntoloViz is based on the packages Plotly (Plotly Technologies Inc. 2021) and Openpyxl (Openpyxl Contributors 2021), which are licensed under the MIT license. OntoloViz is also available through the Python Packaging Index (PyPi) repository and can be installed and run by executing the following commands:

   pip install ontoloviz

   ontoloviz

### 2.2 Usage

#### 2.2.1 Creating the input data

Users can either load a disease or a drug list based on structured ontologies, such as MeSH, or alternative ontologies, like ATC, following a distinct categorization methodology into OntoloViz to generate a graphical visualization. OntoloViz currently supports tab-separated (.tsv) and Excel (.xlsx) files as input data with templates being available in the GitHub repository. The minimum information the user has to provide is either a list of disease or drug names. Colors for display of individual terms can either be manually defined in the input files using the hexadecimal color codes (e.g. #FFFFFF for white) or automatically generated based on manually specified numerical parameters (e.g. term frequencies). Data columns holding additional descriptions for individual disease or drug terms can be provided optionally. The user can also load ontologies from the web based on the .obo format via the “Load from web” button and can configure the templates in an equivalent way as described above.

#### 2.2.2 Configuring the ontology sunburst plots

**Figure 1.**
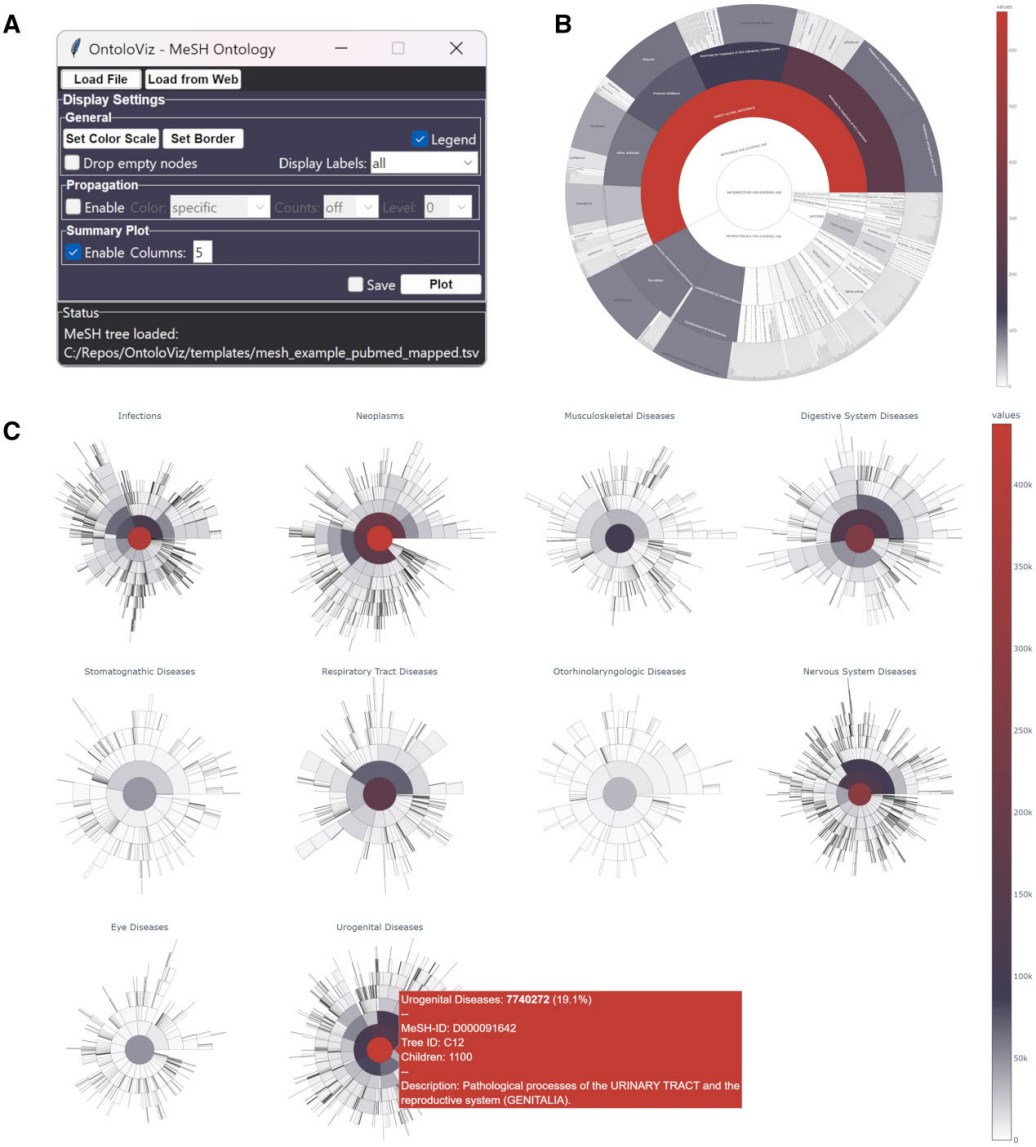
(A) OntoloViz GUI displaying settings for generation and customization of phenotype sunburst plots. (B) Sunburst visualization of COVID-19 clinical trial counts for drugs in the “Anti-infectives for systemic use” branch. Color coding reflects the number of performed COVID-19 clinical trials for each compound. (C) Sunburst visualization of diseases based on publication counts. Counts from child terms have been up-propagated to their parent terms.

Once a template is loaded into OntoloViz, the GUI allows for customization of the generated sunburst plots. Users can define the thresholds for automatic color coding via the “Set Color Scale” menu. Users can furthermore specify the appearance of borders via the “Set Border” menu. Display of node labels in the ontology sunburst visualization can be specified in the “Display labels” menu. By checking the “Drop empty nodes” radio button, empty leaf nodes without annotation counts as specified in the input file will not be displayed in the sunburst visualization. Activating the “Enable propagation” function results in values of child terms to be propagated to their parent terms in the ontology up to the specified level, which in turn also affects color coding and final ontology sunburst visualization. Figure 1A shows the GUI with its configuration options.

#### 2.2.3 Viewing and exporting the ontology sunburst plots

OntoloViz provides two options for viewing the generated sunburst plots. The default option is the summary plot view displaying the full ontology in a single view. The user can specify how many individual sunbursts will be displayed per row. Deselecting the summary plot view allows inspecting individual second level categories of the two ontologies. The created plots can be saved as interactive self-contained .html files by selecting the “Save” option in the GUI, or as static images by clicking the “Save Image” icon in the upper right area of the plot displayed in the browser.

### 2.3 The MeSH controlled vocabulary thesaurus

MeSH, a controlled and structured vocabulary, maintained by the National Library of Medicine (Lipscomb 2000), includes 16 major categories and covers topics such as organisms, chemicals, psychology, anatomy and information science, amongst others. For the examples included in the package, we used the MeSH subtree C (“Diseases”) for our application which holds 23 narrower terms (e.g. neoplasms). In total, there are 4933 unique disease terms within the MeSH disease subtree.

MeSH data were retrieved in XML format from (https://nlmpubs.nlm.nih.gov/projects/mesh/2022/xmlmesh/) in November 2022. Information on MeSH qualifiers, descriptors, and supplemental concept data were extracted to construct the template file available in the GitHub repository.

### 2.4 The ATC classification system

The ATC classification system is a hierarchically structured vocabulary for drugs maintained by the WHO Collaborating Centre for Drug Statistics Methodology. There are 14 main anatomical categories in ATC declared as level 1 (e.g. anti-infectives for systemic use), which ultimately contain the individual compounds or combinations of compounds across five levels. In total, there are 4363 unique pharmaceuticals recorded in the ATC classification system (World Health Organization 1990).

ATC data were retrieved from the ChEMBL SQL database file available at (https://ftp.ebi.ac.uk/pub/databases/chembl/ChEMBLdb/releases/chembl_29/) (Gaulton *et al.* 2017). Information on ATC codes were extracted to construct the template file available in the GitHub repository.

## 3 Applications

### 3.1 Visualization of experimentally tested drugs for COVID-19

For the first example, regarding the “drug” use case, we extracted the list of experimentally tested compounds for COVID-19 from the DrugBank COVID-19 dashboard available at https://go.drugbank.com/covid-19. Information from the table “Clinical Trial Summary by Drug” was used to extract the number of trials for each compound registered at clinicaltrials.gov (National Library of Medicine 2023) naming COVID-19 as condition. Compound names were mapped to their respective ATC names based on information in the ChEMBL database and the number of clinical trials was used for visualizations. The input data for this use case are available in the GitHub repository as “covid_drugs_trial_summary.tsv”. The input data file was loaded into OntoloViz for exploration of compounds tested for COVID-19 in clinical trials.

Figure 1B shows all experimentally tested compounds in the context of COVID-19 belonging to the category of “Anti-infectives for systemic use.” In the context of COVID-19, 88 of the 407 (21.6%) compounds from this category have been investigated in at least one clinical trial. The highest number of clinical trials were conducted for compounds in the third ATC level, “direct acting antivirals,” with 44 of a total of 75 compounds or compound combinations associated with COVID-19.

### 3.2 Visualization of disease-specific publication counts

As example for the “phenotype” use case, we determined the annotation degree for each disease in scientific literature. We therefore downloaded the full set of ∼34.6 million publications from MEDLINE and extracted their associated MeSH terms. All MeSH terms of the subtree C (“Diseases”) being flagged as “major” or holding a respective “major” disease qualifier were included in the analysis. Publication counts were used for color-coding in the sunburst visualization. In this use case we enabled the propagation function, thus propagating individual disease publication counts to the corresponding parent disease terms in the ontology. Data are stored in the template “pubmed_documents_mapped_to_mesh.tsv” in the OntoloViz GitHub repository.

Figure 1C shows the annotation degree based on associated publications for a subset of 10 selected major disease categories. The disease category with the largest number of publications is “Urogenital Diseases” (1100 child terms) with 7.74 million publications followed by “Neoplasms” (1050 child terms) with 7.68 million publications and “Infections” (1386 child terms) with 6.98 million publications.

## 4 Conclusion

In summary, we have developed OntoloViz, an easy-to-use GUI for visualizing ranked lists of diseases or drugs based on hierarchical ontologies such as the MeSH or ATC ontology. Our tool facilitates the visual exploration of vast drug and disease landscapes especially for scientists without any programming experience.

## Data Availability

The source code, test data, template, and documentations are available at GitHub (https://github.com/Delta4AI/OntoloViz).
